# Independent risk and protective factors for oxaliplatin-induced hypersensitivity reactions: a retrospective study

**DOI:** 10.3389/fphar.2025.1624322

**Published:** 2025-07-08

**Authors:** Jing Xue, Zhihao Lian, Xiaoyan Li

**Affiliations:** ^1^ Department of Pharmacy, The Sixth Affiliated Hospital, Sun Yat-Sen University, Guangzhou, China; ^2^ Guangdong Provincial Key Laboratory of Colorectal and Pelvic Floor Disease, The Sixth Affiliated Hospital, Sun Yat-Sen University, Guangzhou, China; ^3^ Biomedical Innovation Center, The Sixth Affiliated Hospital, Sun Yat-Sen University, Guangzhou, China; ^4^ School of Pharmacy, Guangdong Pharmaceutical University, Guangzhou, China

**Keywords:** oxaliplatin, colorectal cancer, hypersensitivity reaction, risk factors, protective factors

## Abstract

**Introduction:**

Oxaliplatin (OXA) serves as a first-line treatment for digestive system tumors such as colorectal cancer (CRC) and gastric cancer. OXA-induced hypersensitivity reactions (HSRs) may pose life-threatening risks to patients. This study aimed to explore the risk factors and protective factors of OXA-induced HSRs in Chinese CRC patients.

**Methods:**

A retrospective analysis was conducted on 233 CRC patients who received OXA treatment between June 2022 and December 2022. Demographic data and medical histories were extracted from the hospital’s medical record system.

**Results:**

Among the 233 patients, 51 patients (21.9%) developed OXA-induced HSRs, with the median treatment cycle at onset being the 4th cycle. Univariate and multivariate analyses revealed that an OXA treatment interruption lasting ≥30 days (P < 0.05, odds ratio [OR] = 10.76, 95% confidence interval [CI] = 4.57–25.3), a history of platinum-based drug allergy (P < 0.05, OR = 19.03, 95% CI = 1.66–217.99), and abnormal absolute neutrophil count (P < 0.05, OR = 8.96, 95% CI = 3.11–25.86) were independent risk factors. Pretreatment with dual-drug or triple-drug prophylactic regimens before OXA administration was identified as an independent protective factor (P < 0.05, OR = 0.37, 95% CI = 0.17–0.82). The area under the receiver operating characteristic (ROC) curve was 0.82 (P < 0.001, 95% CI = 0.75–0.89). Although previous platinum-based drug dosage and abnormal absolute lymphocyte count showed significant differences in univariate analysis, they did not emerge as independent influencing factors in multivariate logistic regression.

**Conclusion:**

Prolonged OXA treatment interruption, a history of platinum-based drug allergy, and abnormal neutrophil count are independent risk factors for OXA-induced HSRs, while dual/triple-drug pretreatment acts as an independent protective factor. Clinicians should evaluate these risks before medication and consider intensified pretreatment regimens to reduce HSR incidence.

## 1 Introduction

Colorectal cancer (CRC) ranks among the most prevalent malignant tumors globally. Despite declining incidence and mortality in many Western countries, the global burden—especially among young adults—continues to rise, resulting in approximately 1–2 million annual deaths (Morgan et al., 2023). OXA-containing regimens are standard first-line therapy for CRC, as recommended by the National Comprehensive Cancer Network (NCCN) (Cheng et al., 2023). As a third-generation platinum compound, OXA binds to DNA, forming cross-links that inhibit replication and transcription, leading to cell cycle arrest and apoptosis (Devanabanda and Kasi, 2025; Raymond et al., 1998).

Hypersensitivity reactions (HSRs), occurring in 8.9%–23.8% of OXA-treated patients (Castells et al., 2012; Cortijo-Cascajares et al., 2013; Mis et al., 2005), are a notable adverse effect that can be life-threatening, often necessitating treatment discontinuation and switching to alternative regimens with potentially inferior efficacy, tolerability, or cost (Tham et al., 2015). Treatment interruptions due to HSRs may compromise therapeutic outcomes, reducing disease-free survival and overall survival (Tham et al., 2015). A complete OXA course (≥6 cycles) is critical for reducing CRC postoperative recurrence, but HSR-induced interruptions may undermine this benefit. With increasing tumor incidence and OXA utilization, HSR-related risks and mortality have garnered growing attention (Gao et al., 2024; Rassy et al., 2023).

While prior studies have associated OXA-induced HSRs with factors such as gender, age, treatment cycles, platinum exposure history, drug interval, infusion duration, and eosinophil count (Parel et al., 2014; Seki et al., 2011; Selcuk and Yıldız, 2023; Sugihara et al., 2012), findings in CRC patients remain inconsistent and controversial. This study aimed to identify independent risk factors and protective factors associated with OXA-induced HSR, and to explore the occurrence characteristics and clinical patterns of it, providing evidence for proactive management.

## 2 Materials and methods

### 2.1 Study population

This is a single-center, observational retrospective study and analysis. For inclusion criteria, patients were eligible if they were diagnosed with CRC, received chemotherapy regimens containing OXA and completed at least one OXA infusion, and had complete and retrievable medical records. OXA can be used in combination with folinic acid and fluorouracil (5-FU) and/or irinotecan (IRI) in the FOLFOX (OXA, folinic acid and 5-FU) or FOLFOXIRI(IRI, OXA, folinic acid and 5-FU) regimens. The administration dose is 85 mg/m^2^ per administration, and it is intravenously administered once every 2 weeks. Additionally, OXA can be used in combination with capecitabine (CAP) in the XELOX (CAP and OXA) regimen, with a dose of 130 mg/m^2^ per administration and intravenous administration once every 3 weeks. Furthermore, the above regimens can be combined with targeted drugs, immunotherapeutic drugs, or tyrosine kinase inhibitors (TKI). For exclusion criteria, patients were excluded if they had a simultaneous diagnosis of other primary tumors, suffered from severe underlying diseases such as severe hepatic and renal failure, intestinal obstruction, or any conditions deemed unsuitable for study inclusion by the researchers, or had missing or incomplete medical records. In this study, the medications used for preventing HSRs before OXA administration are three types: glucocorticoids (dexamethasone), histamine H1 receptor blocking drugs (diphenhydramine and promethazine). According to different combinations, they can be divided into three categories. Single-drug pretreatment refers to the use of any one of the above three drugs, dual-drug pretreatment refers to the combination of glucocorticoids and one of the histamine H1 receptor blocking drugs, and triple pretreatment refers to the combined regimen of simultaneously using the above three drugs. Among them, the dual-drug pretreatment regimen is used in accordance with the recommendations of the guideline (Benson et al., 2024), and the triple-drug pretreatment regimen is based on the clinical treatment plan of this medical institution. This study was approved by the Ethics Committee of the Sixth Affiliated Hospital of Sun Yat-sen University in Guangzhou, China (2023ZSLYEC-297).

### 2.2 Data collection

Demographic and management data, as well as the usage of OXA and drugs for preventing HSRs, were obtained from the electronic medical records of all patients who received OXA treatment in the hospital. Information such as tumor staging (TNM staging), the purpose of chemotherapy, and the status of tumor metastasis was also collected. Additionally, data including serum creatinine, serum neutrophil count, serum eosinophil count, serum lymphocyte count, serum monocyte count, and albumin level were retrieved. Occurrence time of HSRs and patient clinical manifestations were collected.

### 2.3 Definition of outcomes

The primary outcome was OXA-induced HSRs. HSRs were graded according to the National Cancer Institute’s Common Terminology Criteria for Adverse Events (5th edition) (CTCAE 5.0). Determine whether OXA-induced HSR has occurred by assessing whether the patient exhibits typical HSR characteristics. Mild to moderate reactions (grades 1 and 2) were characterized by skin flushing, itching, fever, chills, mild dyspnea, or mild hypotension, or mild gastrointestinal symptoms. Severe reactions (grade 3 and above) typically manifested as bronchospasm, dyspnea, hypotension, and cardiac insufficiency that required treatment (Lenz, 2007). In this study, serum creatinine values were used to reflect renal function. Which embodiment the short-term effect of OXA on renal function. According to the reference ranges of our hospital’s laboratory, the abnormal thresholds for each test index are as follows: Absolute neutrophil count: <2.0 × 10^9^/L or >7.0 × 10^9^/L; Absolute lymphocyte count: <0.8 × 10^9^/L or >4.0 × 10^9^/L; Absolute monocyte count: <0.1 × 10^9^/L or >1.0 × 10^9^/L; Absolute eosinophil count: >0.7 × 10^9^/L; Albumin: <40.00 g/L or >55.00 g/L; Serum creatinine: <44.00 μmol/L (indicating excessive renal function clearance) or >133.00 μmol/L (indicating renal insufficiency).

### 2.4 Statistic analysis

Preliminary observations revealed that the incidence rate of OXA-induced HSRs was approximately 12%. OR can measure the intensity of the association between exposure factors and diseases. Our preliminary estimate found that, the association strength OR between no OXA interval and OXA-induced HSR is approximately 3.78. To ensure an adequate sample size, this study hypothesizes OR = 5, that is, the risk of developing HSR in patients without OXA with an interval of ≥30 days is five times that in patients with an interval of <30 days. A significance level set at 0.05, a statistical power of 0.9, and a ratio of the non-allergy group to the case group of 5:1, we calculated the sample size. Based on these parameters, the required sample size for the control group was determined to be 170 cases, while the sample size for the case group was 34 cases, resulting in a total sample size requirement of 204 cases. However, to enhance the reliability of the study, we ultimately included 233 patients.

We used Excel to summarize the basic information of the patients, meticulously recording details such as gender, age, body mass index (BMI), body surface area (BSA), pretreatment measures, previous dosage of platinum-based drugs, the duration of the interval without OXA, relevant test indicators, clinical manifestations, and grading of allergic reactions. According to the occurrence of HSRs, the cases were classified into the allergic group (n = 51) and the non-allergic group (n = 182), and all variables were categorized as either categorical or continuous variables.

All the original data were statistically analyzed using IBM SPSS Statistics 27 software. We first conducted univariate analysis to preliminarily screen the variables that might trigger HSRs. For categorical variables, the test method was selected based on the theoretical frequency. When the theoretical frequency was ≥5, we employed the chi-square test; when it was <5, Fisher’s exact test was used. For continuous variables, if the data followed a normal distribution, the t-test was applied; if the data exhibited a skewed distribution, the Mann-Whitney U test was utilized. After testing, we found that all the collected continuous variables did not conform to the normal distribution, so we adopted the Mann-Whitney U test. Based on the criterion that P < 0.1 for the test results of all variables, we screened out the variables with significant distribution differences between the allergic group and the non-allergic group and included them in the subsequent multivariate logistic regression analysis.

Moreover, we performed multivariate logistic regression analysis to identify the independent risk factors. Through the “forward stepwise selection method”, we eliminated irrelevant variables and controlled confounding factors. Finally, we retained the variables with P < 0.05 as independent influencing factors. By combining with the OR, we determined whether the variables were protective or risk factors and quantified the degree of influence of each factor. We used the receiver operating characteristic (ROC) curve to assess the performance of the final multivariate model. The multiple logistic regression model was internally validated using 10-fold cross-validation.

## 3 Results

### 3.1 Demographic characteristics

**FIGURE 1:**
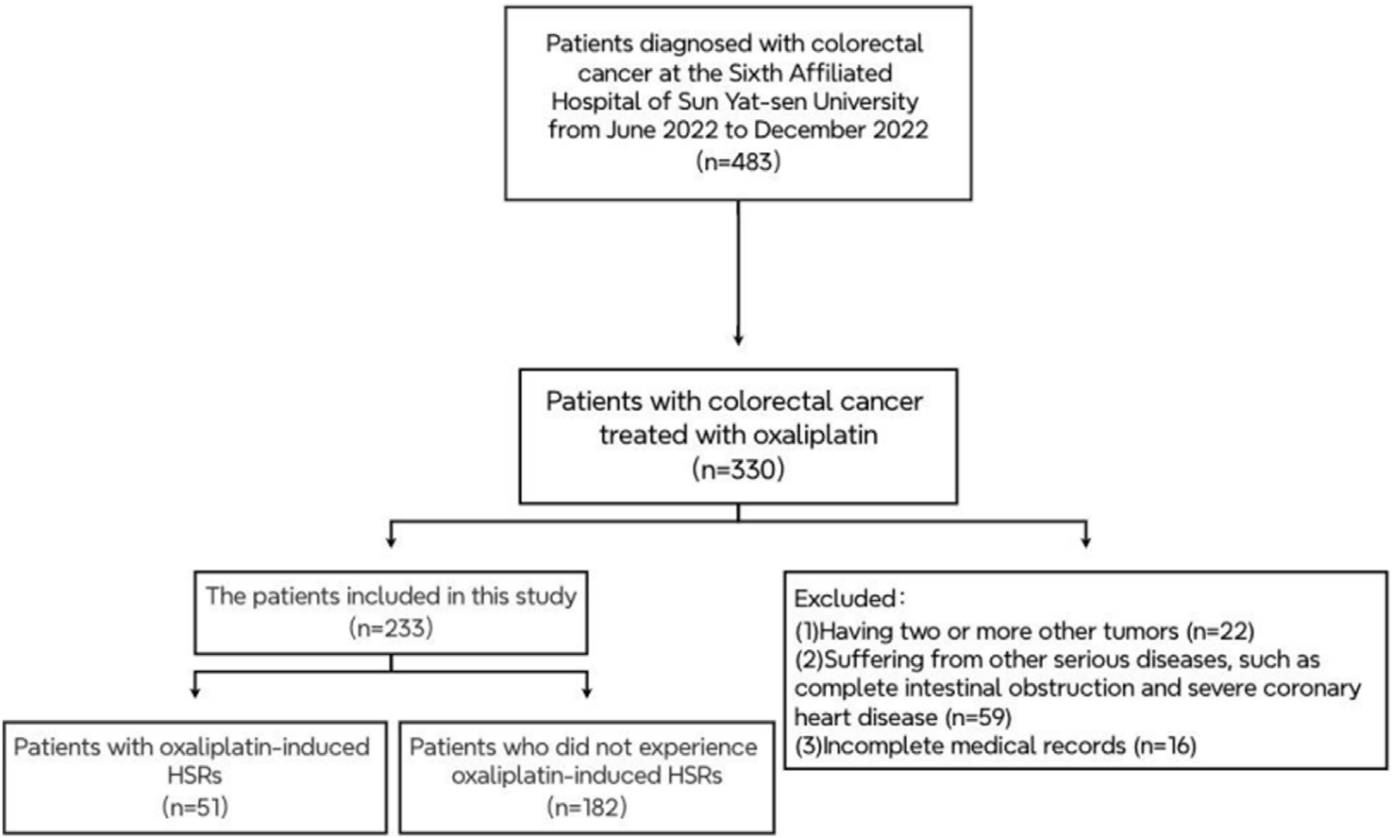
The patient exclusion process in the retrospective study.

Between June and December 2022, an initial search of electronic medical records identified 483 patients diagnosed with CRC, of whom 330 were undergoing OXA treatment. Following a thorough review, 97 cases were excluded, leaving 233 cases for analysis (Figure 1). The baseline characteristics of these patients are presented in Table 1. The median age of the cohort was 58 years (ranging from 24 to 78 years), with 92 patients (39.5%) aged 60 years or older. Among the 233 patients, 140 were male (60%) and 93 were female (40%). Additionally, 74 patients (31.7%) had evidence of liver metastasis.

**TABLE 1:** Comparison of basic information between the hypersensitivity group and the non-hypersensitivity group.

Patient characteristics	Non-hypersensitivity group	Hypersensitivity group	OR	P
Basic information of patients				
Gender (%)			0.62 (0.33–1.16)	0.135
Male	114 (81.4)	26 (18.6)		
Female	68 (73.1)	25 (26.9)		
Age/[M (Q1, Q3), years old]	58 (51, 65)	58 (53, 66)	1.01 (0.99–1.04)	0.714
BMI/[M (Q1, Q3)]	22 (20.2, 23.9)	22 (20.3, 25.4)	1.07 (0.98–1.17)	0.12
BSA/[M (Q1, Q3), ㎡]	1.61 (1.48, 1.72)	1.63 (1.49, 1.72)	1.558 (0.264–9.21)	0.625
Situation of tumor treatment				
Purpose of treatment (neoadjuvant/adjuvant/palliative)	55/74/53	5/30/16		
Liver Metastasis (Yes/No)	54/128	20/31	1.529 (0.8–2.92)	0.197
Information on oxaliplatin Use				
Manufacturer (Aiheng/Lexadin)	167/15	46/5	1.21 (0.42–3.51)	0.725
Dosage of OXA/[M (Q1, Q3), mg]	145 (130, 150)	140 (124, 160)	1 (0.99–1.01)	0.814
Course of treatment/[M (Q1, Q3), times]	3 (2, 6)	4 (2, 7)	1.09 (0.97–1.23)	0.144
Pretreatment situation (None/Single Drug/Dual Drugs or Triple Drug)				<0.05
None	16 (80)	4 (20)		0.03
Single drug	113 (0.86)	19 (14)	0.47 (0.14–1.55)	0.217
Dual drugs or triple drug	53 (65)	28 (35)	0.318 (0.163–0.621)	<0.05
*Previous dosage of platinum-based drugs/[M (Q1, Q3), mg]	0 (0, 0)	280 (0, 1,000)	1.001 (1.001–1.002)	<0.05
*History of allergy to previous platinum-based drugs (Yes/No)	1/181	4/47	15.4 (1.68–141.08)	<0.05
Number of days of interval without OXA ≥30 days (Yes/No)	16/166	24/27	9.22 (4.35–19.56)	<0.05
Inspection information				
Abnormal creatinine (Yes/No)	6/176	4/47	2.5 (0.68–9.21)	0.17
Abnormal albumin (Yes/No)	89/92	31/20	1.6 (0.85–3.02)	0.145
Abnormal absolute neutrophil count (Yes/No)	9/173	14/37	7.27 (2.93–18.06)	<0.05
Abnormal absolute lymphocyte count (Yes/No)	9/173	13/38	6.58 (2.62–16.49)	<0.05
Abnormal absolute monocyte count (Yes/No)	9/173	4/47	1.64 (0.48–5.55)	0.43
Abnormal absolute eosinophil count (Yes/No)	4/178	1/50	0.89 (0.1–8.14)	0.918

Notes: The platinum types in “^*^” refer to OXA.

### 3.2 Occurrence of HSRs

#### 3.2.1 Grading of HSRs

Out of the 233 patients enrolled in the study, 51 patients (21.9%) experienced OXA-induced HSRs. The median treatment cycle at which these HSRs manifested was the 4th cycle. The HSRs were categorized according to severity as follows: Grade 1 reactions were noted in 15 cases, accounting for 29.4% of the HSR cases; Grade 2 reactions were present in 28 cases, making up 54.9%; Grade 3 reactions were observed in 2 cases, representing 3.9%; Grade 4 reactions occurred in 6 cases, accounting for 11.8%. Notably, no instances of Grade 5 reactions were detected during the study period. The specific symptoms and their frequencies of occurrence for these HSRs are detailed in Table 2.

**TABLE 2:** Severity grading and clinical manifestations of allergic reactions in patients.

Severity grade	Number of cases	Proportion (%)	Clinical manifestations (number of cases)
Grade 1	15	29%	Generalized itching (5), hand/foot itching (5), buttock itching (1), chest tightness (1), cold sweating (2), nausea (2), abdominal pain* (1)
Grade 2	28	55%	Facial flushing (17), rash (13), chest tightness (6), dyspnea (3), cold sweating (2), nausea (1), abdominal pain* (3), chills (3), fever (1), high fever (3)
Grade 3	2	4%	Rash (1), facial flushing (1), chest tightness (1), profuse sweating (1), vomiting (1), abdominal pain* (1), hypotension (2)
Grade 4	6	12%	Palmar itching (1), facial flushing (1), chest tightness (2), profuse sweating (5), hypotension (6), abdominal pain* (1), abdominal discomfort (1), anaphylactic shock (6), syncope (1)
Total	51	100%	—

Notes: “*” after “abdominal pain” indicates specific clinical details or annotations (to be supplemented as needed in the original context). It is related to the severity classification of “abdominal pain” in CTCAE, 5.0.

Translated terms follow standard medical terminology (e.g., “anaphylactic shock”, “dyspnea”, “syncope”).

Formatting retains the original structure for clarity, with symptoms listed in parentheses followed by case counts.

An interesting pattern was observed regarding the time of onset in relation to the severity of the reactions. Grade 3 and above HSRs tend to occur relatively early, usually within 10–30 min after administration. In contrast, grade 1 and grade 2 reactions can occur at any time during the entire medication process, even after the medication is completed. Among the patients who developed HSR within 30 min, the proportion of grade 3 and 4 reached 28.6%, while the proportion of patients who developed grade 3 and 4 HSR after 30 min was only 6.7%. We took 30 min as the cut-off time for the onset of HSR and compared the onset time of grade 1–2 and above grade 3 HSR. However, the result did not reach statistical significance (P = 0.052). The reason might be that the sample size was small, resulting in deviations in the statistical values. However, considering the distribution of clinical cases, this result still has certain value. The detailed results are shown in Table 3.

**TABLE 3:** The correlation between the occurrence time and degree of HSRs caused by OXA.

The time from the initiation of OXA use to the occurrence of HSRs	Classification of HSRs		P
	Level 1 and level 2	Level 3 and above	
≤30 min	15	6	0.052
>30 min	28	2	

#### 3.2.2 Related and protective factors of OXA-induced HSRs

Univariate analysis was carried out to identify potential risk factors for OXA-induced HSRs and to explore the associations between patient characteristics and HSR occurrence (Table 1). The analysis revealed that several factors were significantly linked to an elevated risk of OXA-induced HSRs. These included pretreatment with dual or triple anti-allergic drugs prior to OXA administration (odds ratio [OR] = 0.318, P < 0.05), a history of platinum allergy (allergy history to OXA) (OR = 15.4, P < 0.05), the cumulative dose of platinum-based drugs (OR = 1.001, P < 0.05), an OXA treatment interruption lasting ≥30 days (OR = 9.22, P < 0.05), abnormal absolute neutrophil count (OR = 7.27, P < 0.05), and abnormal absolute lymphocyte count (OR = 6.58, P < 0.05). Conversely, factors such as gender, age, BMI, BSA, the presence or absence of liver metastasis, the manufacturer of OXA, creatinine level, and albumin level did not demonstrate a significant influence on the incidence of OXA-induced HSRs.

**FIGURE 2:**
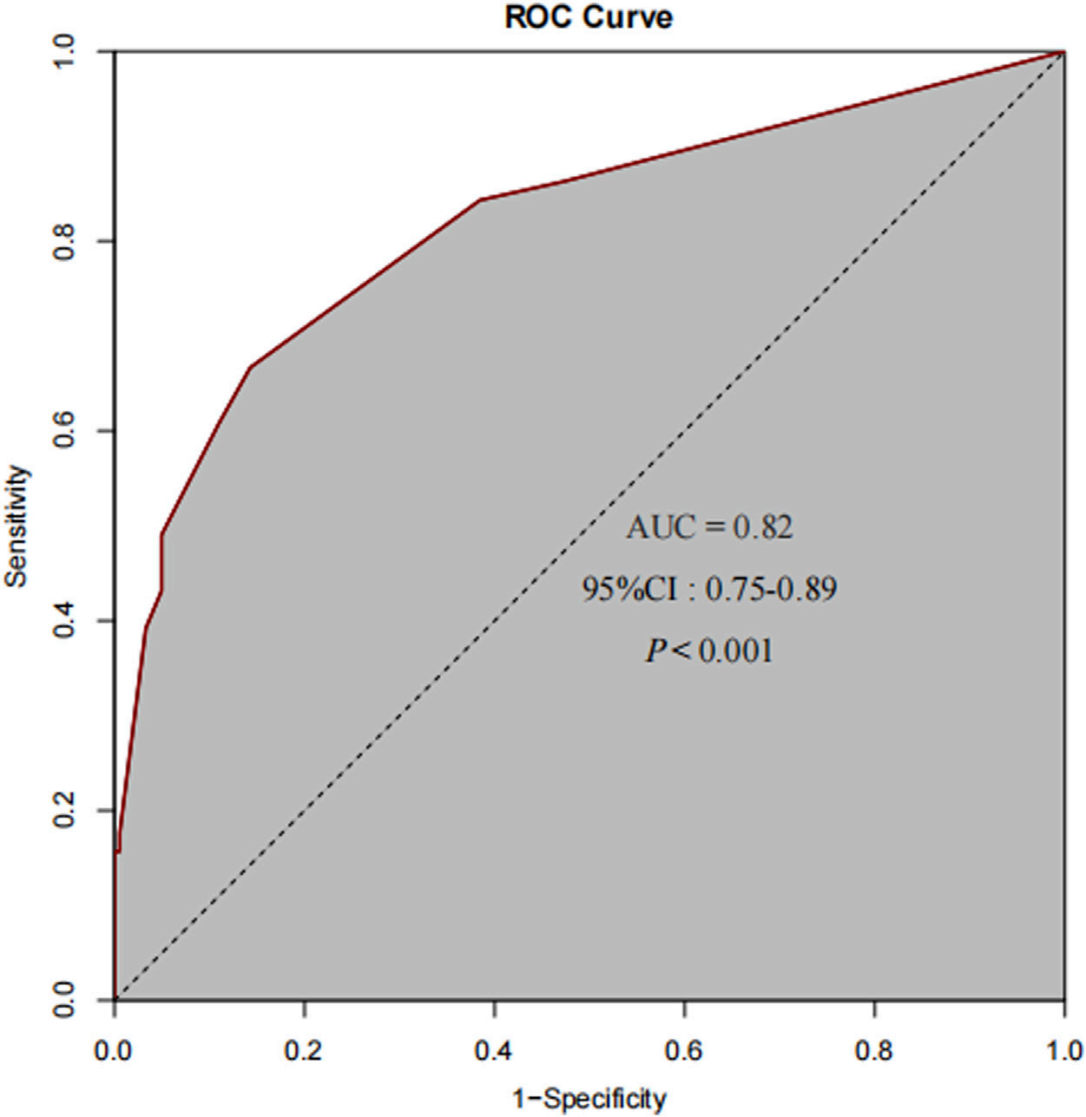
The ROC curve for the final predictive model demonstrates an AUC of 0.82.

Multivariate logistic regression analysis was performed, incorporating six variables: pretreatment strategy, history of platinum-based drug allergy, previous dosage of platinum-based (OXA) drugs, prolonged OXA treatment interruption (≥30 days), abnormal absolute neutrophil count, and abnormal absolute lymphocyte count. The analysis revealed that prolonged OXA treatment interruption (≥30 days) (P < 0.05, OR = 10.76, 95% CI = 4.57–25.3), a history of platinum-based drug allergy (P < 0.05, OR = 19.03, 95% CI = 1.66–217.99), and an abnormal absolute neutrophil count (P < 0.05, OR = 8.96, 95% CI = 3.11–25.86) emerged as independent risk factors for OXA-induced HSRs. Conversely, pretreatment with a dual or triple - drug regimen was identified as an independent protective factor (P < 0.05, OR = 0.37, 95% CI = 0.17–0.82). Specifically, patients with OXA treatment interruption lasting ≥30 days faced an 18.62 - fold increased risk of developing HSRs. Those with a history of platinum-based drug allergy had a 17.65 - fold higher risk, while individuals with an abnormal absolute neutrophil count exhibited a 12.18 - fold elevated risk. In contrast, patients who received dual or triple - drug pretreatment experienced a 68% reduction in the risk of developing HSRs (Table 4). This study attempts to perform a stratified analysis of dual-drug and triple-drug pretreatment regimens to more accurately evaluate their relative protective effects. However, the small sample size of patients receiving triple-drug pretreatment made it difficult to meet the requirements for variable distribution in multivariate logistic regression, leading to data separation. Therefore, after balancing statistical validity with clinical rationality, dual and triple pretreatment approaches were categorized into a single group for statistical analysis. The predictive performance of the final model was evaluated using the receiver operating characteristic (ROC) curve. The area under the ROC curve (AUC) was 0.82 (P < 0.001, 95% CI = 0.75–0.89) (Figure 2). The multivariate logistic regression model underwent internal validation using 10-fold hierarchical cross-validation to assess its generalizability. Results demonstrated that the average AUC across 10 validations was 0.806, closely aligning with the original model’s AUC of 0.82, which indicated strong generalizability. The Hosmer-Lemeshow test yielded a P-value of 0.056 (P > 0.05), confirming acceptable model calibration where predicted probabilities accurately reflected true risk profiles. These findings suggest that the three identified independent risk factors and one independent protective factor can effectively predict the likelihood of OXA-induced HSRs, providing valuable insights for clinical risk assessment and patient management.

**TABLE 4:** Multivariate logistic regression analysis of related factors of HSRs caused by OXA.

Variable	Regression coefficient	Standard error	Wald	P	OR	Lower limit of 95% CI	Upper limit of 95% CI
Number of days of OXA withdrawal >30 days	2.92	0.75	15.39	<0.05	10.76	4.57	25.3
Pretreatment with two or three drugs	−1.13	0.429	6.99	<0.05	0.37	0.17	0.82
History of allergy to platinum-based drugs	2.87	1.22	5.52	<0.05	19.03	2.66	217.99
Abnormal absolute neutrophil count	2.5	1.01	6.11	<0.05	8.96	3.11	25.86
Abnormal absolute lymphocyte count	−0.25	1	0.63	0.8	0.778	0.11	5.53
Previous dosage of platinum-based drugs	−0.001	0.001	1.29	0.26	0.99	0.99	1

## 4 Discussion

OXA is a non - cycle - specific antitumor agent. By inhibiting DNA replication and transcription, it induces cell cycle arrest and apoptosis, making it a widely adopted first-line treatment for CRC (Devanabanda and Kasi, 2025). Among its adverse reactions, HSRs are particularly notable. Currently, clinical practice lacks systematic management strategies, diagnostic criteria, and treatment protocols for OXA-induced HSRs. Although these reactions are often mild, they can occasionally progress to severe forms, potentially leading to patient mortality (Aroldi et al., 2015). Despite the well - recognized risk of OXA - induced HSRs in CRC patients, the underlying factors contributing to their development remain incompletely understood. This study aimed to fill this gap by conducting a retrospective analysis of CRC patients treated with OXA. The results identified three novel independent risk factors: prolonged OXA treatment interruption (≥30 days), a history of platinum allergy, and abnormal neutrophil count. Additionally, pretreatment with dual or triple drugs was found to be an effective protective strategy.

This study discovered a significant association between prolonged OXA treatment interruption, defined as ≥30 days, and the occurrence of OXA-induced HSRs, establishing it as a critical risk factor. Our finding aligns with prior research (Mori et al., 2010; Sohn et al., 2018; Song et al., 2022), indicating that an extended OXA-free interval is closely linked to HSR development. From an immunological perspective, the body’s immune system may undergo changes during the extended OXA - free period. It is possible that the immune cells, which have been previously exposed to OXA, become sensitized. Once OXA is re - administered, these sensitized immune cells can recognize the drug as a foreign substance and initiate an immune response (Pichler, 2022). For example, T - lymphocytes, which play a key role in cell - mediated immunity, may be primed during the treatment interruption. When re - exposed to OXA, they can activate other immune cells such as macrophages and eosinophils, leading to the manifestation of HSRs. This is supported by studies on drug - induced immune responses, which have shown that a break in drug exposure can alter the immune memory and subsequent responses (Pichler, 2022). In clinical settings, most patients discontinuing OXA for 30–45 days do so due to colorectal tumor surgery. Surgery itself is a major stressor that can suppress the immune system in the short - term (Kim, 2018). This postoperative immunosuppression, combined with the re - introduction of OXA after a long - term interruption, creates a high - risk situation for HSRs (Ezanno et al., 2022). Therefore, the relationship between prolonged OXA treatment interruption and HSRs is a complex one and has significant clinical significance. Further research is needed to fully understand the underlying mechanisms and formulate more effective patient management strategies.

Notably, this study determined that an abnormal absolute neutrophil count serves as an independent risk factor for HSRs, diverging from prior studies that implicated eosinophils (Palapinyo et al., 2022; Seki et al., 2011). This discrepancy may stem from methodological differences: our study categorized neutrophil counts as binary variables (i.e., normal vs. abnormal), emphasizing clinically significant deviations, whereas most prior research analyzed count data as continuous variables. Studies have pointed out that neutrophils are crucial in both the sensitization and induction stages of contact hypersensitivity (Felix et al., 2015). Neutrophils can exacerbate local inflammation by releasing neutrophil extracellular traps, and this mechanism has been confirmed in drug allergic reactions (Song et al., 2016). However, most previous studies on the relationship between blood cells and OXA-induced HSRs have focused on eosinophils. Eosinophils are known to be involved in allergic reactions, as they can release cytotoxic proteins and lipid mediators that contribute to tissue damage and the inflammatory response. In contrast, the current study’s finding of neutrophils as an independent risk factor for HSRs may be due to differences in research design. Moreover, other research in different disease contexts has shown that neutrophil abnormalities can have significant impacts. For example, in cancer studies, an elevated neutrophil count in the peripheral blood of liver cancer patients has been identified as an independent risk factor affecting survival (Margetts et al., 2018; Xu et al., 2023). In summary, the identification of abnormal absolute neutrophil count as an independent risk factor for OXA-induced HSRs in this study challenges previous views. Further research is needed to clarify the underlying mechanisms, perhaps through more in-depth exploration of neutrophil function in the context of OXA exposure, and by comparing different neutrophil-related parameters (such as functional assays rather than just count abnormalities) across studies.

The occurrence of OXA-induced HSRs is positively correlated with a patient’s history of allergy to platinum-based drugs. This study’s findings revealed that patients with such a history face a significantly elevated risk of HSRs, with the risk increasing by 17.65 - fold. All platinum-based drugs can bind to proteins in the human body, forming platinum-protein complexes. These complexes are recognized as antigens by the immune system. Due to the structural similarities among platinum-based drugs, there is a risk of re-allergy upon re-exposure (Gao et al., 2024). When re-exposed to platinum-based drugs, these substances bind to IgE antibodies on the surface of mast cells. This binding triggers mast cell degranulation, leading to the release of inflammatory mediators such as histamine and leukotrienes, which in turn initiate type I HSRs. Clinically, a history of allergy to platinum-based drugs indicates that the patient’s immune system has heightened sensitivity to these agents. As a result, subsequent contact with platinum-based drugs is likely to trigger another rapid-onset type I HSR. Therefore, for patients with a history of platinum allergy, it is crucial to exercise extreme caution and remain highly vigilant against the recurrence risk of HSRs.

The combined use of two or three drugs prior to OXA administration serves as an independent protective factor against OXA-induced HSRs, which aligns with the outcomes of previous research (Okayama et al., 2015; Pagani et al., 2022; Yoshida et al., 2015). Thus, prior to the clinical application of OXA, for patients at high risk of developing HSRs, such as those with an interval in platinum-based drug treatment or a history of platinum-based drug allergy, intensified pretreatment should be considered, and a dual- or triple-drug prophylactic regimen can be employed. The European EAACI guidelines advocate for dual-drug pretreatment, specifically the combination of glucocorticoids and antihistamines (Pagani et al., 2022). Yoshida et al. (2015) proposed in their study that pretreatment with corticosteroid drugs can prevent the occurrence of OXA-induced HSRs. Okayama et al. (2015) through a retrospective analysis, discovered that prophylactic dual - drug pretreatment is effective for patients with mild HSRs and can be regarded as a first-line approach for re-introducing the drug. Our study also found that dual-drug or triple-drug pretreatment can significantly lower the risk of HSRs, further validating the protective effect of pretreatment. Nevertheless, determining whether triple-drug pretreatment is more effective than dual-drug pretreatment necessitates a prospective randomized controlled study with a larger sample size.

Grade 3 and above HSRs are more likely to occur within 30 min after OXA administration. This discovery is consistent with the existing research results (Mori et al., 2010; Sohn et al., 2018). Grade 3 or above HSRs that occur within 30 min may be a type I hypersensitivity reaction. When patients are exposed to OXA again, the cross-linking of IgE antibodies can trigger rapid degranulation of histamine, trypsin and pro-inflammatory cytokines, leading to severe systemic reactions (such as allergic reactions) (Pichler, 2019). This is supported by studies showing that immediate-type HSRs (onset <1 h) correlate with higher serum tryptase levels and IgE-mediated mechanisms (Bavbek et al., 2022). Therefore, it is strongly recommended that clinicians remain vigilant and closely monitor patients for any suspected symptoms of HSRs within 30 min post - administration.

It is noteworthy that the prevalence of oxaliplatin-induced hypersensitivity reactions (HSRs) may vary significantly across different ethnic groups. The incidence of HSRs in Western populations ranges from 8.9% to 23.8% (Castells et al., 2012; Cortijo-Cascajares et al., 2013; Mis et al., 2005), while the prevalence in Chinese patients in this study was 21.9%. Although overlapping with Western data, the genetic background of Asian populations may play an important role. For example, the distribution of human leukocyte antigen (HLA) alleles is race-specific (Cao et al., 2001), and HLA gene polymorphisms are one of the core mechanisms leading to immune differences. These alleles may increase the risk of HSRs by affecting T-cell recognition of oxaliplatin-protein complexes (Joerger, 2012; Ledys et al., 2018; Li et al., 2021). In addition, CYP3A4 gene polymorphisms in Asian patients may lead to faster metabolism of glucocorticoids (Eeftinck Schattenkerk and Lager, 2013; Wang et al., 2016),which led to insufficient anti-inflammatory effect of the standard pretreatment regimen, might explain why the protective effect of two-drug or three-drug pretreatment was more significant in this study. These differences suggest that future studies should include multi-ethnic cohorts to clarify the association between HLA typing, immune system differences, and HSRs, so as to develop individualized prevention strategies.

## 5 Limitations

Through this retrospective study, the key factors of OXA-induced HSRs have been revealed, but there are still some limitations. Firstly, there are limitations in the sample. This study is only a single-center and retrospective study. The patients come from a single source, and the tumor types are mainly CRC. The sample size is relatively small, and the data rely on adverse drug reaction (ADR) reporting records and medical records. There may be the lack of some information, which may lead to biases in the research results. Secondly, according to the literature reports, OXA-induced HSRs are very likely to have an impact on the overall survival of patients. However, since the overall survival information of patients could not be obtained in this paper, the correlation between OXA-induced HSRs and overall survival was not discussed. Again, as a single-center study of the Chinese population, it is impossible to assess the impact of racial differences on HSR. Therefore, in the future, multi-center and multi-ethnic studies need to be carried out to clarify genetic susceptibility (such as HLA typing) and the racial adaptability of OXA-induced HSRs.

## 6 Conclusion

Prolonged OXA interruption, platinum allergy history, and abnormal neutrophil count are independent risk factors for HSRs, while dual/triple-drug pretreatment is protective. For patients who have discontinued OXA for more than 30 days, enhanced prevention and close monitoring are required when OXA is used again. Particular attention should be paid to suspected HSRs symptoms within 30 min after OXA administration. Studies with a larger sample size are needed to verify these findings.

## Data Availability

The raw data supporting the conclusions of this article will be made available by the authors, without undue reservation.

## Abbreviations

OXA, Oxaliplatin; CRC, Colorectal Cancer; 5- FU, 5-Fluorouracil; IRI, Irinotecan; CAP, Capecitabine; LV, Leucovorin Calcium; TKI, Tyrosine Kinase Inhibitor; FOLFOX, 5-FU+LV+OXA; FOLFOXIRI, 5-FU+LV+OXA+IRI; XELOX, CAP+OXA; HSR, Hypersensitivity Reaction; ROC, Receiver Operating Characteristic Curve; ADR, Adverse Drug Reaction; EAACI, European Academy of Allergy and Clinical Immunology; CTCAE, Common Terminology Criteria for Adverse Events; AUC, Area Under the Curve; DNA, Deoxyribonucleic Acid.
